# Non-invasive assessment of skeletal muscle fibrosis in mice using nuclear magnetic resonance imaging and ultrasound shear wave elastography

**DOI:** 10.1038/s41598-020-78747-8

**Published:** 2021-01-11

**Authors:** Aurea B. Martins-Bach, Damien Bachasson, Ericky C. A. Araujo, Lucas Soustelle, Paulo Loureiro de Sousa, Yves Fromes, Pierre G. Carlier

**Affiliations:** 1 AIM & CEA NMR Laboratory, Neuromuscular Investigation Center, Institute of Myology, Paris, France; 2 Neuromuscular Physiology Laboratory, Neuromuscular Investigation Center, Institute of Myology, Paris, France; 3 Université de Strasbourg, CNRS, ICube, FMTS, Strasbourg, France

**Keywords:** Diagnostic markers, Neuromuscular disease, Magnetic resonance imaging, Ultrasonography

## Abstract

Fibrosis is a key pathological feature in muscle disorders, but its quantification mainly relies on histological and biochemical assays. Muscle fibrosis most frequently is entangled with other pathological processes, as cell membrane lesions, inflammation, necrosis, regeneration, or fatty infiltration, making in vivo assessment difficult. Here, we (1) describe a novel mouse model with variable levels of induced skeletal muscle fibrosis displaying minimal inflammation and no fat infiltration, and (2) report how fibrosis affects non-invasive metrics derived from nuclear magnetic resonance (NMR) and ultrasound shear-wave elastography (SWE) associated with a passive biomechanical assay. Our findings show that collagen fraction correlates with multiple non-invasive metrics. Among them, muscle stiffness as measured by SWE, T_2_, and extracellular volume (ECV) as measured by NMR have the strongest correlations with histology. We also report that combining metrics in a multi-modality index allowed better discrimination between fibrotic and normal skeletal muscles. This study demonstrates that skeletal muscle fibrosis leads to alterations that can be assessed in vivo with multiple imaging parameters. Furthermore, combining NMR and SWE passive biomechanical assay improves the non-invasive evaluation of skeletal muscle fibrosis and may allow disentangling it from co-occurring pathological alterations in more complex scenarios, such as muscular dystrophies.

## Introduction

Fibrosis is observed in almost any tissue in response to injury or within pathological processes. It is characterized by loss of tissue function due to an accumulation of extracellular matrix, predominantly composed of collagen^1^. In skeletal muscles, diffuse or endomysial fibrosis develops in various conditions, including diabetes and aging, but is particularly relevant in neuromuscular disorders such as muscular dystrophies. In these diseases, skeletal muscle fibrosis can be observed even before the detection of muscle degeneration^2^ and has been associated with worse prognoses^3^ and reduced effect of possible therapies^4^.

While other skeletal muscle alterations such as atrophy, fat infiltration, and inflammation may be non-invasively assessed with NMR^5^, quantitative methods to evaluate skeletal muscle fibrosis non-invasively are still lacking. Currently, fibrosis is mainly quantified by histological and biochemical assays. However, these methods require biopsies, invasive procedures that poorly reflect the whole muscle status.

Imaging modalities including nuclear magnetic resonance (NMR) and ultrasound (US) evaluate the whole muscle non-invasively and may be sensitive to fibrosis^6–13^. In the heart, myocardial fibrosis has been related to increased extracellular volume (ECV) estimated with NMR^6,7^. In skeletal muscles, thickened perimysial layers can be imaged with NMR^11^, but it depends on high spatial resolution, currently unavailable in clinical scanners. Other NMR variables, such as T_1_, T_2_, and T_2_*, may also be altered by fibrosis in skeletal muscle^8–10^, although it is unclear how and to which extent. Fibrosis has also been associated with increased muscle stiffness in passive biomechanical assays^14,15^. Ultrasound shear-wave elastography (SWE) allows real-time assessment of tissue stiffness and has been considered a reliable marker for staging hepatic fibrosis^13^, being potentially sensitive to skeletal muscle fibrosis.

The use of NMR and SWE to evaluate skeletal muscle fibrosis is however challenging. Skeletal muscle fibrosis most frequently develops simultaneously with other pathological processes such as membrane lesions, inflammation, necrosis, regeneration, and fat infiltration, which may affect both NMR and SWE metrics, possibly impairing fibrosis estimation.

In this study, we aimed at assessing how collagen fraction, an invasive marker of skeletal muscle fibrosis, correlates with non-invasive NMR and SWE metrics when other pathological processes are minimized. For that, we developed a non-dystrophic mouse model with variable levels of skeletal muscle fibrosis while minimizing inflammation, fat infiltration, and membrane leakage. Mice were evaluated in vivo by NMR [native-T_1_ (T_1_ before injection of Gd contrast agent), extracellular volume (ECV), T_2_, T_2_*, perfusion, and texture analysis on T_1_-weighted images] and SWE (muscle shear modulus at variable muscle length). Correlations between NMR/SWE metrics and collagen fraction obtained from histological analysis were investigated, in addition to partial correlation analyses where perfusion metrics from NMR and inflammation, calcification, and centronucleation scores from histology were included as covariates. The sensitivity and specificity of non-invasive NMR and SWE metrics in the identification of fibrotic muscles were then evaluated.

## Results

### Fibrosis was successfully induced in healthy skeletal muscles

**Figure 1:**
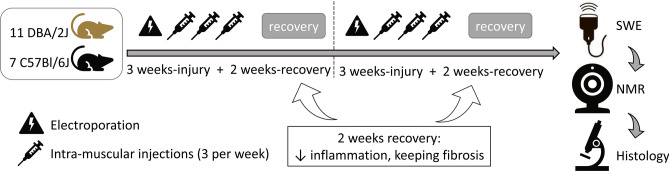
Experimental design: fibrosis induction in wild-type mice. Extensive (electroporation) and localized injuries (intramuscular injections in the left tibialis cranialis (TC)) were combined on the first day of the two injury cycles. Localized injuries were then repeated three times per week during 3 weeks. A 2-week interval at the end of each injury cycle allowed the reduction of muscle inflammation, while fibrosis was still present. The injury cycle took place twice. Only the left leg was injured, and muscles in the right leg served as controls for each mouse in paired comparisons. After 10 weeks of injury/recovery, nuclear magnetic resonance (NMR) and shear-wave elastography (SWE) were performed in vivo. Muscles were then collected after euthanasia for histological analysis. DBA/2J and C57Bl/6 were included in this study to induce variable levels of skeletal muscle fibrosis, as DBA/2J mice are more susceptible to develop fibrosis than C57Bl/6 mice due to a polymorphism in the *Ltbp4* gene^16,17^.

Two genetic backgrounds, C57BL/6 and DBA/2J, were selected to allow the induction of variable levels of fibrosis in wild-type mice. DBA/2J mice harbour a polymorphism in the *Ltbp4* gene that increases the susceptibility to develop fibrosis when compared to C57BL/6 mice^16,17^. A combination of one extensive injury (electroporation of the left leg) and repeated localized injuries [intramuscular injection of saline solution thrice weekly in the left tibialis cranialis (TC)] was applied twice. A 2-week interval after each injury cycle allowed tissue regeneration and reduction of the inflammation levels while fibrosis still developed. Uninjured contralateral TCs (right) served as control muscles in pairwise comparisons (Fig. 1).

**Figure 2:**
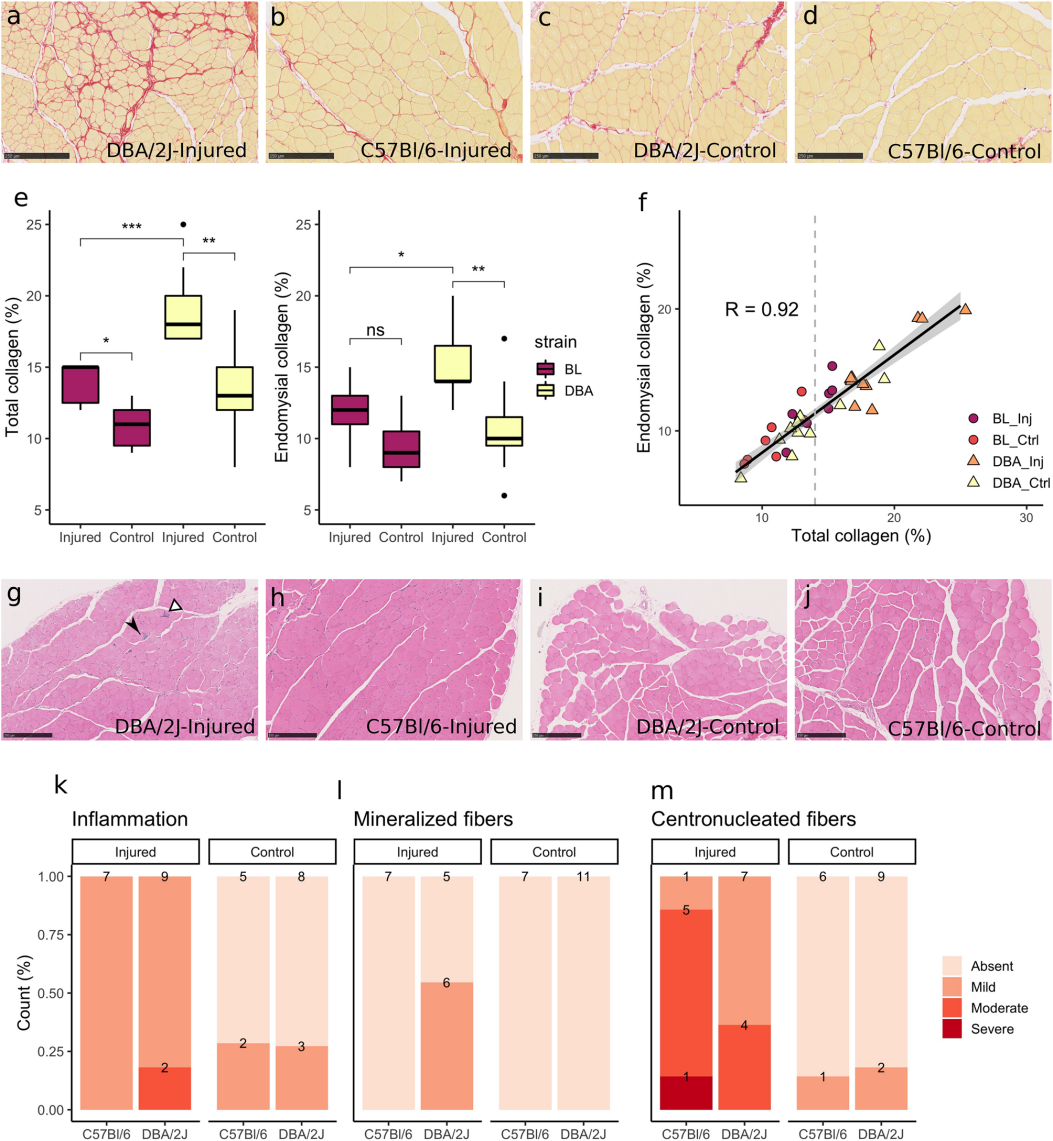
Injury induced fibrosis in non-dystrophic muscles. (**a**–**d**) Sirius red collagen staining of (**a**) DBA/2J injured muscle, showing marked fibrosis in red; (**b**) C57BL/6 injured muscle, with a moderate increase in collagen staining; (**c**) DBA/2J and (**d**) C57BL/6 non-injured control muscles, showing thin connective tissue layers characteristics of normal skeletal muscles. (**e**) Boxplots show increased total and endomysial collagen fraction in injured muscles, especially in DBA/2J mice. (**f**) Total and endomysial collagen fractions were highly correlated (R = 0.92). The majority of non-injured samples presented total collagen fraction below 14%, while the injured muscles presented collagen fraction higher than 14% in general. (**g**–**j**) Haematoxylin–eosin staining of (**g**) DBA/2J injured muscle, with moderate inflammation (white arrowhead) and presence of mineralized fibers (black arrowhead); (**h**) C57BL/6 injured muscle, showing high count of centronucleated fibers but no inflammation; (**i**) DBA/2J and (**j**) C57BL/6 non-injured control muscles. (**k**–**m**) Increased collagen fraction occurred simultaneously with mild inflammation (**k**), low count of necrotic mineralized fibers in DBA/2J mice (**l**) and regeneration, reflected by the presence of centronucleated fibers (**m**). (**a**), (**c**): magnification × 10, scale bar 250 μm. *p < 0.05, **p < 0.01, ***p < 0.001, *ns* non-significant.

This combination of extensive and localized repeated injuries successfully induced endomysial skeletal muscle fibrosis in wild-type mice (Fig. 2a–f), with approximately 40% increase in total and endomysial collagen fraction in injured muscles. Total collagen surface fraction in Sirius red stains was 17 (3)% in injured muscles and 12.5 (2)% in control muscles (delta: 4 (4)%, p = 0.0004; Fig. 2e). Endomysial collagen fraction was 14 (2)% versus 10 (3)% in injured and controls muscles, respectively (delta: 3 (4)%, p = 0.002; Fig. 2e). When each strain was evaluated separately, total collagen fraction was increased in both strains (DBA/2J-injured: 18.0 (3.0)%, DBA/2J-control: 13.0 (3.0)%, delta: 5.0 (4.0)%, p = 0.006; C57BL/6-injured: 15.0 (2.5)%, C57BL/6-control: 11.0 (2.5)%, delta: 3.0 (2.5)%, p = 0.03), and endomysial collagen was increased in DBA/2J mice (DBA/2J-injured: 14.0 (2.5)%, DBA/2J-control: 10.0 (2.0)%, delta: 4.0 (5.0)%, p = 0.006; C57BL/6-injured: 12.0 (2.0)%, C57BL/6-control: 9.0 (2.5)%, delta: 3.0 (2.5)%, p = 0.11).

DBA/2J mice presented higher collagen fraction than C57BL/6 mice in injured TCs, for both total collagen (DBA/2J-Inj: 18 (3)%, C57Bl/6-Inj: 15 (3)%, p = 0.0005) and endomysial collagen (DBA/2J-Inj: 14 (3)%, C57Bl/6-Inj: 12 (2)%, p = 0.02). No differences were observed in non-injured muscles from DBA/2J and C57Bl/6 mice for total collagen fraction (DBA/2J: 13 (3)%, C57Bl/6: 11 (3)%, p = 0.05) and for endomysial collagen fraction (DBA/2J: 10 (2)%, C57Bl/6: 9 (3)%, p = 0.31) (Fig. 2e).

The proposed model induced variable collagen fraction in skeletal muscles, with total collagen ranging from 8 to 25%, and endomysial collagen ranging from 6 to 20% of the tissue surface. Total and endomysial collagen fractions were strongly correlated (R = 0.92, p = 2.8e^−15^, Fig. 2f). Despite the presence of fibrosis in injured muscles, no fat infiltration and only mild to moderate inflammation were observed (Fig. 2g–m). Centronucleated regenerating fibers were common features after injury in both mouse strains (Fig. 2g,h,m). Additionally, mineralized necrotic fibers could be observed in injured DBA/2J muscles (Fig. 2g,l).

### Perfusion is higher in injured skeletal muscles

Muscle perfusion was assessed with NMR-arterial spin labelling (ASL). A hyperaemic response paradigm with ischemia–reperfusion stress was used to increase the global perfusion and highlight differences between normal and altered muscles^18^. Perfusion was measured at rest for 3′ 20″, during 6′ 50″ of ischemia and following the hyperaemic response for 10′ 40″.

The global volume repaid during the hyperaemic response after ischemia (total perfusion) was higher in injured muscles (total perfusion: injured: 252.6 (138.6) ml/100 g, control: 50.0 (161.0) ml/100 g, delta: 172.9 (88.4) ml/100 g, p = 2.3e^−5^), and presented a mild but significant correlation with endomysial collagen (R = 0.35, p = 0.035). When the strains were evaluated separately, both presented higher muscle perfusion in injured muscles (DBA/2J-injured: 271.1 (151.6) ml/100 g, DBA/2J-control: 97.1 (190.5) ml/100 g, delta: 187.4 (63.5) ml/100 g, p = 0.001; C57BL/6-injured: 207.7 (106.5) ml/100 g, C57BL/6-control: 35.0 (142.6) ml/100 g, delta: 137.4 (141.1) ml/100 g, p = 0.047). No difference was detected in maximal perfusion when grouping the two strains (injured: 91.5 (37.0) ml/min/100 g, control: 80.9 (50.4) ml/min/100 g, delta: 14.5 (62.8) ml/min/100 g, p = 0.20) or when the strains were evaluated separately (DBA/2J-injured: 92.2 (38.3) ml/min/100 g, DBA/2J-control: 74.3 (66.5) ml/min/100 g, delta: 19.3 (61.9) ml/min/100 g, p = 0.12; C57BL/6-injured: 77.5 (40.6) ml/min/100 g, C57BL/6-control: 86.0 (25.7) ml/min/100 g, delta: 4.2 (41.5) ml/min/100 g, p = 0.94). Increased total perfusion after injury could be caused by altered vascular control, angiogenesis or vascular remodelling during the regeneration process^19,20^, all possibly affecting other NMR and SWE measurements. To take vascular alterations into account, total and maximal perfusion were considered as covariates in all partial correlation analyses.

### Extracellular volume (ECV) correlates with collagen fraction in skeletal muscles

ECV was estimated from muscle and plasma T_1_ measured before and one hour after an injection of Gd-contrast agent (Gd-CA, Supplementary Fig. S1), according to Eq. (1):

1 $$ECV=\frac{{\left(\frac{1}{{T1}_{postGd}}-\frac{1}{{T1}_{preGd}}\right)}_{muscle}}{{\left(\frac{1}{{T1}_{postGd}}-\frac{1}{{T1}_{preGd}}\right)}_{plasma}}$$

**Figure 3:**
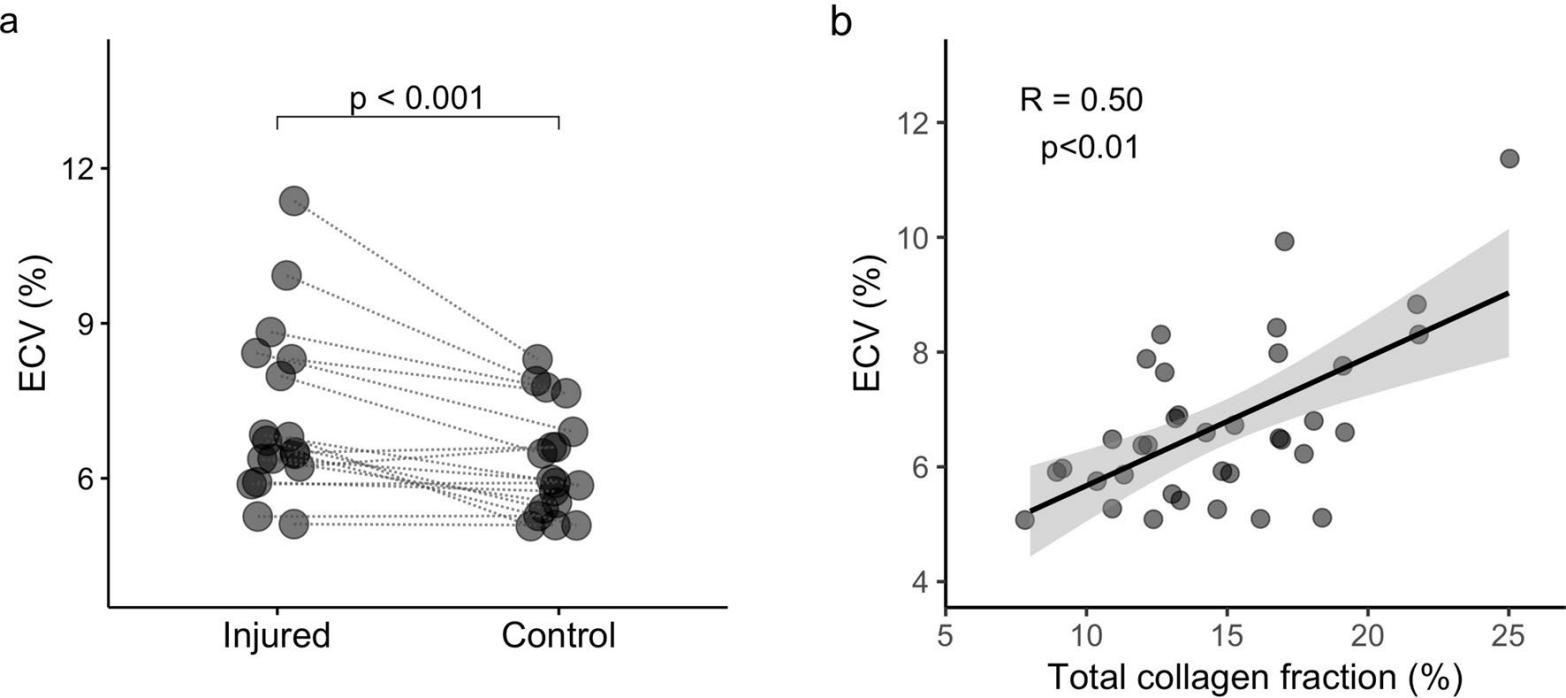
ECV correlates with collagen fraction in skeletal muscles. Extracellular volume (ECV) was estimated from muscle T_1_-maps acquired in vivo and plasma-T_1_ measured in samples collected before and 1-h after Gd-CA injection. ECV was higher in fibrotic muscles after injury (**a**) and correlated with collagen fraction (**b**). Lines connect injured and control muscles for each animal in (**a**). (**b**) Scatter plot with fitted linear regression and 95% confidence interval in grey.

Injured muscles presented approximately 15% higher ECV when all mice were evaluated together (injured: 6.6 (2.0)%, control: 5.9 (1.4)%, delta: 0.9 (1.4)%, p = 0.0008; Fig. 3a). When the strains were evaluated separately, the differences in ECV did not reach significance (DBA/2J-injured: 8.0 (2.1)%, DBA/2J-control: 6.6 (1.9), delta: 1.4 (1.3)%, p = 0.13; C57BL/6-injured: 6.4 (0.6)%, C57BL/6-control: 5.7 (0.4)%, delta: 0.4 (0.8)%, p = 0.07). ECV correlated significantly with collagen fraction (ECV × Collagen-total: R = 0.50, p = 0.002; Fig. 3b). Within the partial correlation analysis, the correlation between ECV and collagen fraction was reduced, but remained significant (ECV × Collagen-total: R′ = 0.38, p = 0.028).

No difference could be detected in native-T_1_ between injured and control muscles when the two strains were evaluated together (injured: 1647.0 (38.6) ms, control: 1649.9 (37.6) ms, delta: − 9.5 (38.6) ms, p = 0.18) or when the strains were evaluated separately (DBA/2J-injured: 1661.6 (29.9) ms, DBA/2J-control: 1657.0 (48.6) ms, delta: − 3.5 (37.1) ms, p = 0.90; C57BL/6-injured: 1628.7 (59.0), C57BL/6-control: 1635.0 (31.1), delta: − 28.2 (35.5) ms, p = 0.46). No correlation between native-T_1_ and collagen fraction could be detected (Supplementary Table S1).

### T_2_-maps revealed a negative correlation between T_2_ and collagen fraction in skeletal muscle

In this study, we used two complementary approaches to estimate T_2_ values: MSME, which allows the estimation of T_2_ values voxel-by-voxel with a high spatial resolution (T_2_-map), but with a possible bias due to diffusion and local susceptibility effects; and ISIS-CPMG, which allows the extraction of a T_2_-spectrum from a volume, with less bias due to diffusion and local susceptibility changes, but with lower spatial resolution.

**Figure 4:**
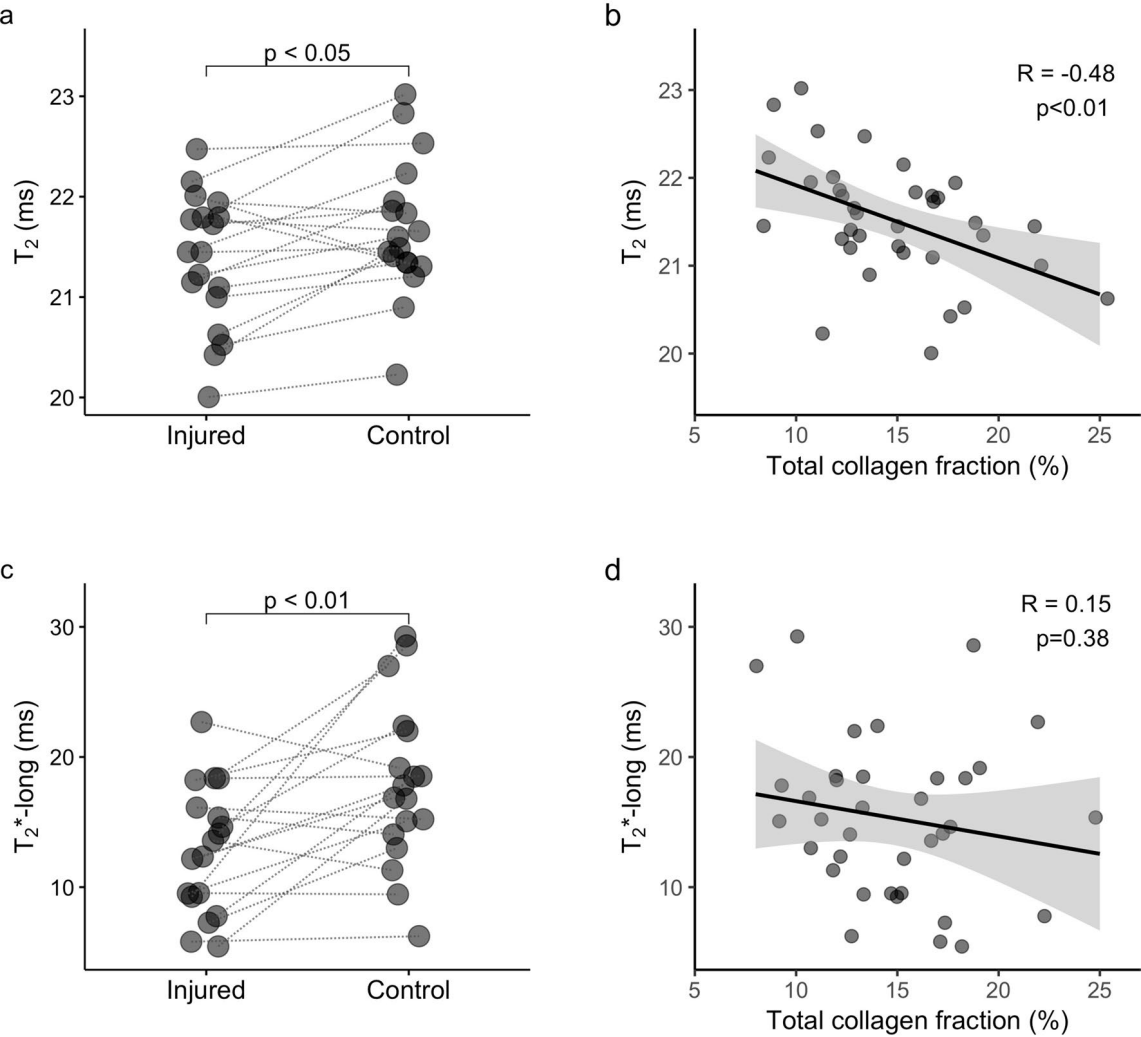
Lower T_2_ and long-T_2_* in fibrotic muscle after injury. (**a**) T_2_ maps calculated from MSME data revealed shorter T_2_ in injured skeletal muscles, which correlated negatively with collagen fraction (**b**). (**c**) T_2_* estimation from UTE signal decay with a bi-exponential off-resonance model showed that water long-T_2_* was also reduced in injured muscles, but it did not correlate with collagen fraction (**d**). Lines connect injured and control muscles for each animal in (**a**) and (**c**). (**b**, **d**) Scatter plot with fitted linear regression and 95% confidence interval in grey.

T_2_-maps estimated from MSME data showed a slightly reduced T_2_ in injured muscles in paired comparisons when both strains were grouped (injured: 21.4 (0.8) ms, control: 21.5 (0.6) ms, delta: − 0.2 (0.7), p = 0.014; Fig. 4a). However, no significant difference was detected when each strain was evaluated separately (DBA/2J-injured: 21.1 (1.2) ms, DBA/2J-control: 21.4 (0.3) ms, delta: − 0.2 (0.3) ms, p = 0.56; C57BL/6-injured: 21.8 (0.7) ms, C57BL/6-control: 22.2 (0.9) ms, delta: − 0.8 (0.6) ms, p = 0.16). T_2_ estimated from MSME data correlated negatively with total collagen fraction (R = − 0.48, p = 0.003; Fig. 4b), and this correlation was stronger when inflammation, centronucleated fibers, calcifications, and perfusion variables were considered as covariates in the partial correlation analysis (R′ = − 0.50, p = 0.003).

ISIS-CPMG data was acquired on a voxel over the tibialis cranialis muscle (mean dimensions 0.9 × 1.0 × 4.6 mm^3^; Supplementary Fig. S2a), and the high signal-to-noise ratio (SNR) allowed to model the signal as a combination of three components with different T_2_ values. The T_2_ values for each component (short-, intermediary- and long-T_2_) and the respective fractions were estimated. However, despite the slight reduction in muscle T_2_ estimated from MSME data, no differences could be observed between injured and control muscles for any of the T_2_ values or fractions extracted from ISIS-CPMG data (Supplementary Table S2). Additionally, T_2_ values and fractions extracted from ISIS-CPMG data did not correlate with collagen fraction (Supplementary Table S1).

### Reduced water T_2_* in injured skeletal muscles

Ultra-short time to echo (UTE) acquisitions using variable echo times allowed the estimation of T_2_* values in the tissue. UTE signal decay was best characterized by a bi-exponential model with a short-T_2_* component chemically shifted by 970 Hz from the water frequency, and a water component with long T_2_*. 970 Hz is the chemical shift difference between water and the methylene group in macromolecules at 7 T^10,21,22^. The source of this signal is still debatable, being possibly originated from lipids^10^, phospholipids, and/or collagen or other macromolecules^21,22^^.^ No fat suppression was used to avoid interfering with this oscillating signal since a contribution from collagen cannot be excluded.

No differences were observed in the short-T_2_* value or in the fractions of each component when the two strains were analysed together (short-T_2_*: injured 0.51 (0.71) ms, control 0.63 (0.88) ms, delta: − 0.11 (0.44), p = 0.26; short-T_2_* fraction: injured 5.8 (4.1)%, control 4.9 (3.5)%, delta: 0.35 (3.01)%, p = 0.61) or separately (short-T_2_*: DBA/2J-injured: 0.86 (1.99) ms, DBA/2J-control: 0.82 (1.13) ms, delta: − 0.14 (1.18) ms, p = 0.90; C57BL/6-injured: 0.39 (0.16) ms, C57BL/6-control: 0.43 (0.26) ms, delta: − 0.08 (0.25) ms, p = 0.46; short-T_2_* fraction: DBA/2J-injured: 5.9 (5.2)%, DBA/2J-control: 4.4 (4.7)%, delta: 0.4 (3.4)%, p = 0.71; C57BL/6-injured: 5.0 (2.7)%, C57BL/6-control: 6.1 (1.6)%, delta: 0.3 (1.8)%, p = 0.70).

Interestingly, water long-T_2_* was reduced in injured muscles (long-T_2_*: injured 13.0 (6.6) ms, control 17.3 (7.0) ms, delta: − 5.1 (8.4), p = 0.005; Fig. 4c). However, no difference was detected in water long-T_2_* when each strain was evaluated separately (DBA/2J-injured: 14.1 (9.3) ms, DBA/2J-control: 18.5 (7.2) ms, delta: − 5.7 (10.2), p = 0.12; C57BL/6-injured: 12.2 (4.7) ms, C57BL/6-control: 16.9 (4.8) ms, delta: − 4.7 (3.7) ms, p = 0.13).

No correlation was observed between water long-T_2_* and collagen fraction (Fig. 4d, Supplementary Table S1). However, there was a mild but significant correlation between endomysial collagen fraction and short-T_2_* in the partial correlation analysis, when histological and perfusion changes were considered as covariates (R′ = 0.36, p = 0.038; Supplementary Table S1).

### Texture analysis in high spatial resolution images

**Figure 5:**
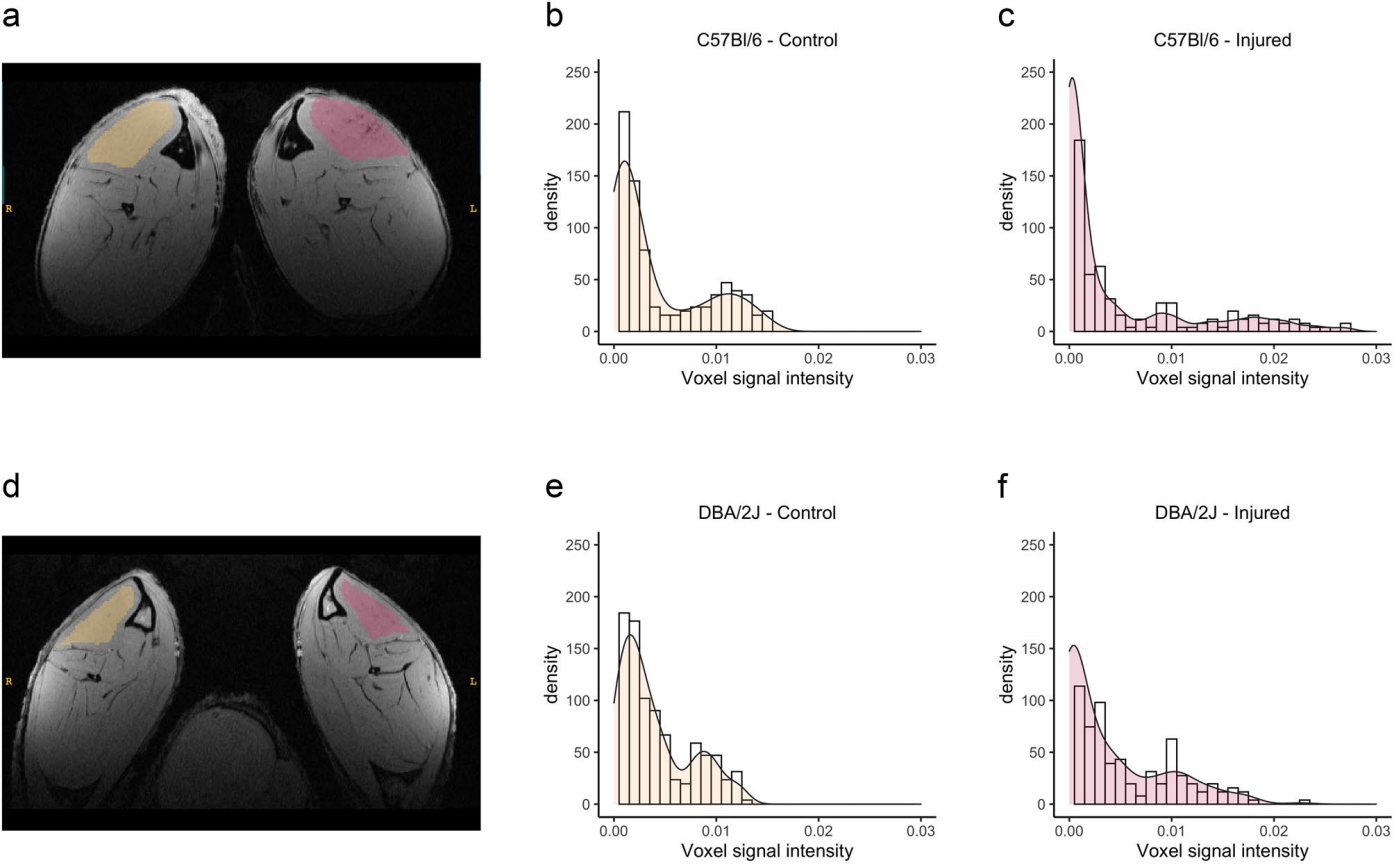
Altered signal intensity distribution in high-resolution images of injured muscles. T1-weighted high-resolution images were acquired with in-plane resolution of 50 μm and slice thickness of 200 μm. Representative high-resolution image of a C57Bl/6 (**a**) and a DBA/2J (**d**) mouse, showing the ROIs drawn over injured (red) and control (yellow) tibialis cranialis and the histograms of signal intensity in the ROIs: (**b**) C57Bl/6—control, (**c**) C57/Bl/6—injured, (**e**) DBA/2J—control, and (**f**) DBA/2J—injured. Both C57Bl/6 (**c**) and DBA/2J (**f**) histograms illustrate the higher kurtosis observed in injured muscles, with increased presence of outliers.

Texture analysis was performed by extracting histogram features from T_1_-weighted gradient-echo (GRE) images acquired at 50 μm in-plane resolution and 200 μm slice thickness. Figure 5 shows representative images of a C57Bl/6 and a DBA/2J mouse, and the histograms of signal intensity in the left (injured) and right (control) tibialis cranialis. Skewness, kurtosis, and energy values for injured and control muscles are detailed in Supplementary Table S3. When the strains were evaluated together, injured muscles exhibited higher kurtosis and energy (kurtosis-injured: 4.7 (2.8), kurtosis-control: 3.6 (0.7), p = 0.03; energy-injured: 11.1 (3.2) × 10^3^, energy-control: 9.5 (2.5) × 10^3^, p = 0.02). When the strains were evaluated separately, C57Bl/6 mice had increased energy in injured muscles (injured: 13.3 (3.4), control: 9.4 (1.7), p = 0.015). Kurtosis correlated with total (R = 0.33, p = 0.046) and endomysial collagen fraction (R = 0.42, p = 0.010), and energy correlated with endomysial collagen fraction (R = 0.35, p = 0.037). When histological and perfusion variables were considered as covariates in the partial correlation analysis, only kurtosis correlated with endomysial collagen fraction (R = 0.36, p = 0.039; Supplementary Table S1).

### SWE detects increased stiffness in fibrotic skeletal muscles

**Figure 6:**
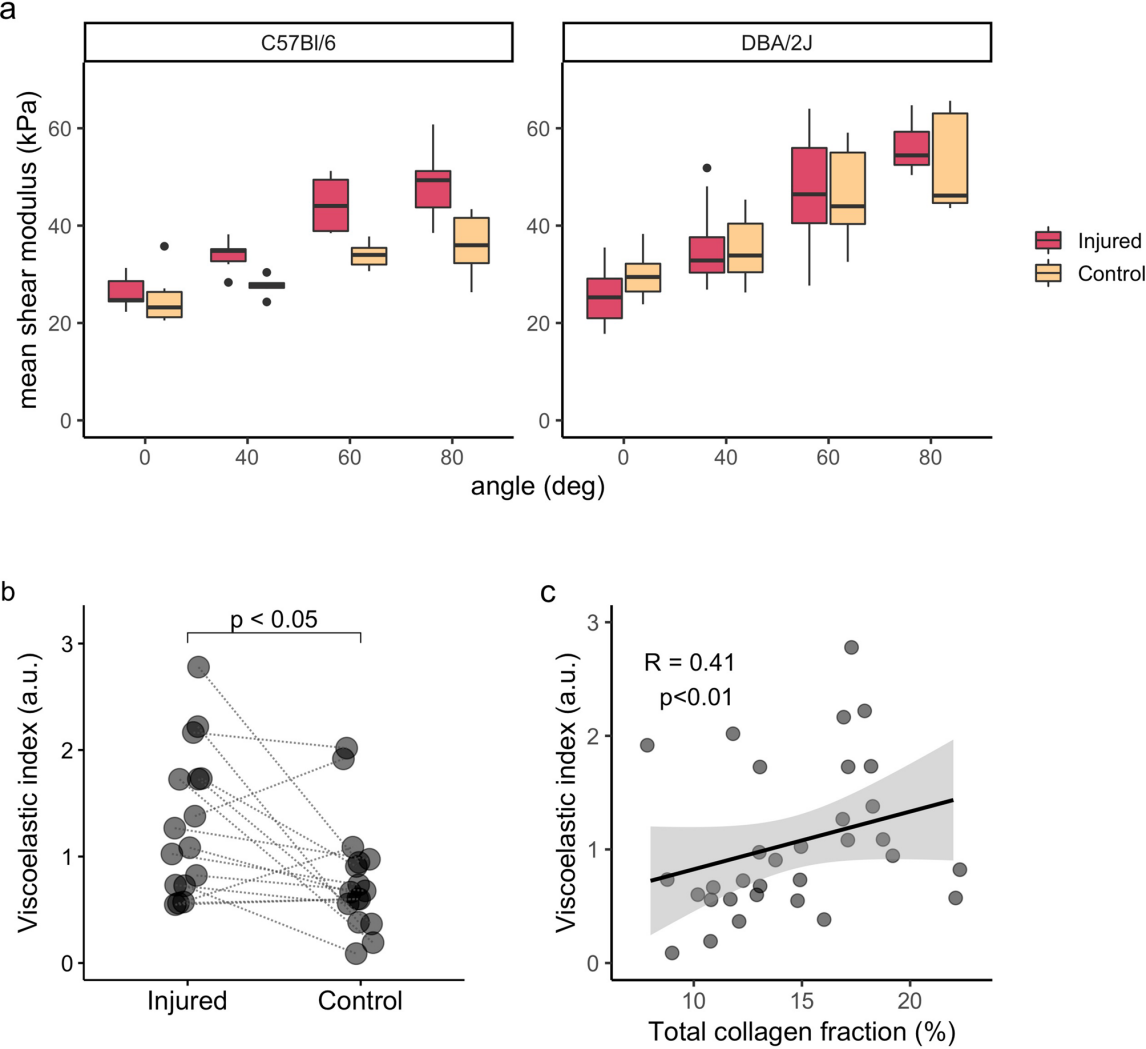
Increased stiffness in fibrotic skeletal muscle. (**a**) Boxplots showing that ankle plantar flexion increases the shear-wave modulus in both injured and control muscles, from C57BL/6 and DBA/2J mice, but no difference between injured and control muscles could be detected (Supplementary Table S4). (**b**) The viscoelastic index was higher in injured muscles and (**c**) correlated with collagen fraction, indicating that muscle stiffness is increased in fibrotic muscles and that it can be detected non-invasively with SWE. Lines connect injured and control muscles for each mouse in (**b**). (**c**) Scatter plot with fitted linear regression and 95% confidence interval in grey.

B-mode ultrasound images and shear modulus (SM) maps were acquired at 0°, 40°, 60°, and 80° of ankle plantar flexion (Supplementary Fig. S3). SM values increased during ankle extension for both strains (Fig. 6a). No significant difference was observed between injured and control muscles when grouping all mice, but when the strains were evaluated separately DBA/2J showed reduced stiffness in injured muscles at rest, and C57BL/6 mice presented increased stiffness at 40° and 60° of plantar flexion (Supplementary Table S4).

A viscosity index, SMi, was calculated according to Eq. (2):

2 $$SMi={\frac{{SM}_{80}-{SM}_{0}}{{SM}_{0}}}$$

with *SM*_*80*_ and *SM*_*0*_ the shear modulus at 80° and 0° of ankle plantar flexion, respectively.

SMi was increased in injured muscles when C57BL/6 and DBA/2J mice were evaluated together (1.17 (1.00) a.u. versus 0.67 (0.44) a.u., delta: 0.29 (0.86) a.u., p = 0.016; Fig. 6b). No difference could be detected when the strains were evaluated separately (Supplementary Table S4).

Both SM_80_ and SMi correlated with total collagen fraction (SM_80_: R = 0.46, p = 0.009, SMi: R = 0.41, p = 0.019, Fig. 6c). SM_80_ correlated with total collagen fraction when perfusion and histological alterations were considered as covariates in the partial correlation analysis (R′ = 0.35, p = 0.044), and both SM_0_ (at rest) and SM_80_ correlated with endomysial collagen fraction in partial correlation analyses (SM_0_: R′ = 0.38, p = 0.031, SM_80_: R′ = 0.35, p = 0.047; Supplementary Table S1).

### Combining non-invasive modalities improves the identification of skeletal muscle fibrosis

To evaluate the ability of NMR and SWE markers to identify skeletal muscle fibrosis, the metrics that correlated with collagen fraction (Supplementary Table S1) were selected for ROC analysis. Since SMi was evaluated, SM_0_ and SM_80_ were excluded from this analysis to avoid redundancies. The collagen fraction in our murine model was lower than the maximum values observed in muscle dystrophy patients^23^, so samples were divided into only two groups. Samples with total collagen fraction up to 14% were classified as normal (22 samples), and those with total collagen fraction from 15 to 25% were classified as fibrotic (14 samples). The threshold of 14% corresponded to the intersection between the median collagen fraction plus the interquartile range for non-injured muscles (12.5% (2.0), and the median minus the interquartile range for injured muscles (17.0% (3.0)) (Fig. 2f).

**Figure 7:**
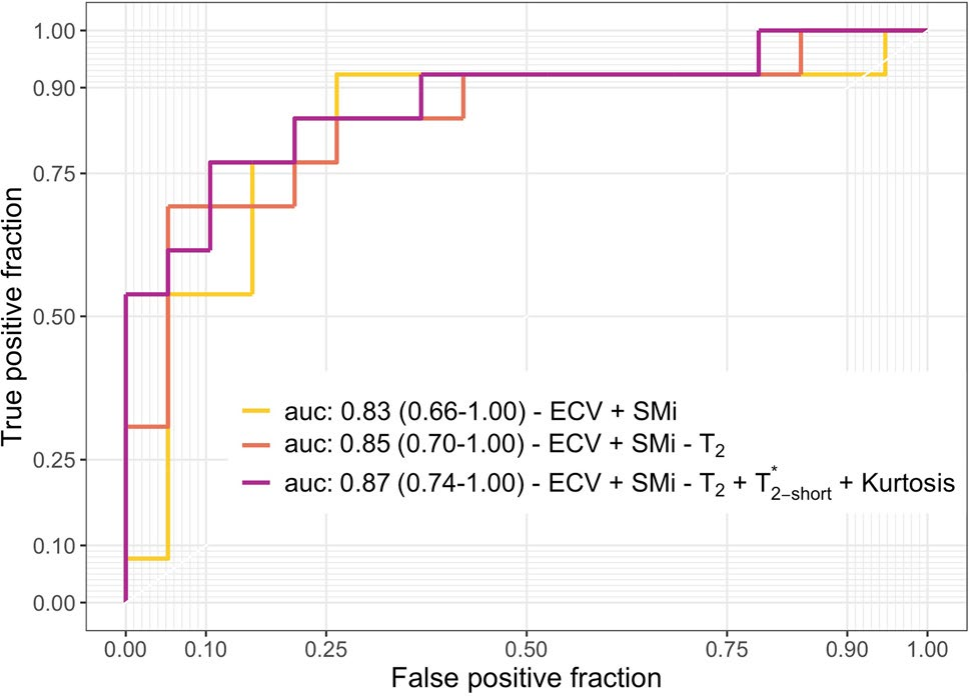
Combining non-invasive metrics to assess skeletal muscle fibrosis. Combining ECV and SMi (light orange line), ECV, SMi and T_2_ estimated from MSME data (red line) or ECV, SMi, T_2_ (MSME), short-T_2_* and Kurtosis (purple line) lead to AUCs always above 0.8. The combination of NMR and SWE metrics improved the ability to distinguish fibrotic (total collagen fraction from 15 to 25%) from non-fibrotic muscles (total collagen fraction up to 14%) non-invasively. Values are presented as AUC (95% confidence interval).

When each metric was evaluated separately, SMi and ECV presented the highest AUCs (Table 1). The evaluated metrics were then gradually added to assess the diagnostic power of composed indexes. The same weight was assigned to all metrics, and each metric could contribute positively or negatively to the composed index. Figure 7 shows that when ECV and SMi were added, an AUC of 0.83 was achieved (95% confidence interval: 0.66–1.00), and when ECV, SMi, and T_2_ (from MSME data) were combined, AUC was 0.85 (95% confidence interval: 0.70–1.00). A maximal AUC of 0.87 was obtained when combining ECV, SMi, short-T_2_*, T_2_ (from MSME data), and Kurtosis (95% confidence interval 0.74–1.00). Including more metrics did not further improve the AUC.

**Table 1:** ROC analysis for NMR and SWE metrics that correlated with collagen fraction.

Modality	Metric	AUC	95% CI
SWE	SMi	0.77	0.60–0.95
NMR-ECV	ECV	0.74	0.56–0.93
NMR-T_2_ map (MSME)	T_2_	0.69	0.51–0.87
NMR-T_2_* map (UTE)	Short-T_2_*	0.63	0.41–0.85
NMR-texture	Kurtosis	0.60	0.40–0.81
	Energy	0.51	0.31–0.71

*AUC* area under the curve, *CI* confidence interval, *SWE* shear-wave elastography, *ECV* extracellular volume.

## Discussion

The present study shows that skeletal muscle fibrosis affects non-invasive metrics, notably leading to higher ECV, lower T_2_ as estimated from MSME data, and increased viscoelastic index. Our murine model presented skeletal muscle fibrosis without fat infiltration or dystrophic lesions, and only minimal inflammation, allowing a better characterization of the effects of fibrosis on NMR and SWE metrics.

Variable levels of fibrosis in skeletal muscles were achieved by combining injuries in aged mice from two genetic backgrounds: while aging leads to an accumulation of extracellular matrix^24^, DBA/2J mice are more susceptible to develop fibrosis than C57Bl/6 due to a polymorphism in the *Ltbp4* gene^16,17^. The presence of fibrosis in skeletal muscles while other pathological processes were minimized makes this murine model a promising resource for studies on skeletal muscle fibrosis and potential therapies^23^.

ECV, estimated from blood- and myocardium-T_1_ pre-/post-injection of Gd-CA, is a well-validated method to assess myocardial fibrosis^6,7^. In cardiac imaging, blood- and myocardium-T_1_ are extracted from the same T_1_-map. This approach is virtually impossible in skeletal muscle imaging due to the smaller blood volume and the anatomy of blood vessels. In our study, skeletal muscle ECV could be estimated by measuring plasma-T_1_ in samples collected before and after Gd-CA injection. It is worth noting that we observed ECV values that were considerably lower than those reported in the myocardium^6,7^, reflecting the smaller microvascular volume fraction and milder fibrotic response in injured skeletal muscles than in post-infarct myocardium.

Several pathogenic mechanisms can affect ECV as measured with NMR: fibrosis, inflammation, and vascular changes can lead to a real expansion of the extracellular space, while membrane lesions allow Gd-CA to distribute inside cells, also resulting in increased ECV measures. In our study, ECV was increased in fibrotic muscles and correlated with collagen fraction when other pathological processes were minimized, indicating that ECV is indeed sensitive to fibrosis in skeletal muscle.

T_2_ estimated with MSME sequence was slightly lower in injured muscles, but no difference was detected in T_2_ values and fractions calculated from ISIS-CPMG data. T_2_ estimated from MSME acquisitions can be biased by diffusion effects combined with local susceptibility changes, while T_2_ estimated with ISIS-CPMG is less affected due to a shorter inter-echo-spacing. Indeed, simulations suggest that skeletal muscle fibrosis might alter NMR-diffusion metrics^25^, while a susceptibility-induced signal drop is observed in perimysial layers on high-resolution gradient-echo images^11,26^. Interestingly, T_2_ estimated with MSME had a stronger correlation with collagen fraction in the partial correlation analysis, further indicating that reduced T_2_ due to fibrosis might be masked by increased T_2_ induced by inflammation, regeneration, and vascular changes.

Injured muscles also presented reduced long-T_2_*, in agreement with observations in fibrotic myocardium^10^. However, long-T_2_* did not correlate with collagen fraction. Kurtosis and energy were also increased in injured muscles in high-resolution gradient-echo T_1_w-images and correlated with collagen fraction. Changes in both long-T_2_* and texture features in gradient-echo images (GRE) reinforce the hypothesis of altered tissue susceptibility, which could be a consequence of fibrosis and/or co-occurring alterations, such as calcifications or vascular changes^19^. Calcifications, blood vessels, and possibly fibrosis lead to changes in local tissue susceptibility and consequently to dephasing and reduced signal intensity, especially in images acquired with GRE sequences. In this study, GRE sequences were used for T_2_* measurements (UTE) and to acquire high-resolution images (FLASH), both being thus similarly affected by local susceptibilities changes. In the high-resolution images acquired in this study, this local alteration in signal intensity may not have been enough to change the mean signal intensity on the whole muscle. However, the distribution of signal intensities in the region of interest was altered, as quantified by changes in kurtosis and energy.

Interestingly, short-T_2_* correlated with collagen fraction, despite not showing differences between injured and control muscles. Short-T_2_* signal with a chemical shift difference of 970 Hz from the water peak has been associated with lipids and phospholipids^10^, but also with collagen and other macromolecules^21,22^. The correlation between the oscillatory short-T_2_* signal and collagen fraction might indicate that this signal is indeed affected by collagen in fibrotic skeletal muscles. How much do collagen density and maturation impact on this signal remains an open question. While tendons present very short T_2_* and no visible oscillatory component^27^, collagen solutions present longer T_2_* values for the component with short-T_2_*, and a clear oscillatory pattern on the signal^21,22^. In fibrotic skeletal muscles, non-compacted and/or immature collagen molecules might be contributing to this oscillatory signal with short T_2_*.

To avoid overfitting, we limited the models used to fit the UTE signal decay to up to two components. There was a clear oscillating component on the signal decay, and F-tests confirmed that the model with an oscillatory component with short-T_2_* best fitted the majority of the data. As a consequence, this choice of model with one oscillating and one on-resonance component prevented us from quantifying any water component with short-T_2_* (water bound to macromolecules). While the contribution of bound water was undetectable with this technique, it does not mean it is negligible, and we acknowledge it as a limitation of our approach.

On the SWE metrics, the viscoelastic index was increased in injured skeletal muscles and correlated with collagen fraction, indicating that SWE can detect increased muscle stiffness induced by fibrosis. It is in agreement with previous studies describing the consequences of fibrosis on passive muscle mechanics, with increased collagen fraction leading to higher muscle stiffness^14,15,28^. Additionally, it agrees with the reported use of SWE to stage liver fibrosis^13^. Using SWE to evaluate fibrotic skeletal muscle is challenging though: muscle stiffness can be altered by factors not related to pathologies, like muscle contraction and changes in the relative probe-muscle positioning. In this study, we used a standardized positioning system for mice and probe, controlling muscle length and without any pressure exerted by the probe. It allowed measuring muscle viscoelastic properties at different muscle lengths and confirmed that passive stretch-induced stiffening correlates with fibrosis in skeletal muscles.

Among the non-invasive metrics evaluated, ECV and SWE presented the highest power to identify fibrotic skeletal muscles. Combining metrics in a composed index further increased the ability to discriminate between fibrotic and non-fibrotic muscles. We hypothesize that the combination of non-invasive metrics might allow to disentangle skeletal muscle fibrosis from co-occurring pathological alterations in more complex scenarios, and further studies are needed to test this hypothesis. Other metrics, such as those extracted from MR-elastography, diffusion-MRI, and quantitative susceptibility mapping, might improve even more the non-invasive assessment of muscle fibrosis, and such a combined index may allow the non-invasive estimation of the collagen fraction in skeletal muscles.

Fibrosis has been associated with increased distance between muscle fibers and capillaries, pointing to a potential reduction in muscle perfusion due to fibrosis^3^. Indeed, hypertensive rats present reduced skeletal muscle perfusion, as a potential consequence of perivascular fibrosis^29^. In our study, however, muscle perfusion was increased in injured muscles, possibly reflecting the angiogenesis and vascular remodelling that take place during muscle regeneration^19,20^. Both angiogenesis and altered vascular control would affect local tissue susceptibility and proportion of free water, impacting on NMR and SWE metrics and being a confounding factor in our model. As a limitation, we could not assess the muscle vascular network with histology. To account for it, perfusion metrics were considered as an indirect measurement of vascular remodelling and included as covariates in partial correlation analyses.

In conclusion, our findings showed that in a mouse model with skeletal muscle fibrosis and minimized concurrent pathological alterations, fibrosis is associated with increased extracellular volume (ECV) estimated with NMR, increased stretch-induced stiffening detectable with shear-wave elastography (SWE), and reduced T_2_ from T_2_ maps. ECV and the viscoelastic index as measured with SWE were the best markers of skeletal muscle fibrosis, and the combination of non-invasive metrics improved the ability to identify fibrotic muscles. This study shows that NMR and SWE are sensitive to skeletal muscle fibrosis when other pathological processes are minimized, a necessary step towards the non-invasive assessment of skeletal muscle fibrosis in complex pathologies, such as muscular dystrophies.

## Materials and methods

### Animal model

Seven C57Bl/6 and eleven DBA/2J male mice were studied with NMR and histology. One C57BL/6 and one DBA/2J mouse were excluded from SWE analyses due to technical issues during data acquisition (six C57Bl/6 and ten DBA/2J mice studied with SWE). These two genetic backgrounds were selected since DBA/2J mice are more prone to develop fibrosis than C57Bl/6 mice due to a polymorphism in the *Ltbp4* gene^16,17^. This study was performed following French laws and regulations concerning the use of animals for research (license #12975 validated by the Ethical Committee Charles Darwin n-5, Paris, France).

Fibrosis was induced in mice’s left tibialis cranialis muscle (TC) by combining two types of injuries. Electroporation of the left leg (8 pulses, 100 V, 20 ms pulse duration, 2 Hz pulse frequency, pulse train applied twice turning the electrodes by 90°) caused extensive muscle injury, followed by repeated local injuries with intramuscular injections of saline solution, thrice-weekly during 3 weeks (NaCl 0.9%, 50 µl). Subsequently, 2 weeks without injuries allowed tissue regeneration and reduction of inflammation. This combination of extensive injury, repeated local injuries, and regeneration was applied twice (10-weeks-long injury protocol). At the endpoint, mice (1-year-old) were evaluated in vivo by SWE and NMR. Tissue was then collected for histology. Uninjured right TCs served as control muscles in pairwise comparisons. Figure 1 illustrates the experimental protocol used to induce diffuse skeletal muscle fibrosis in C57BL/6 and DBA/2J wild-type mice.

### Ultrasound shear-wave elastography

Muscle shear modulus (SM) was measured using an Aixplorer ultrasound scanner driving a 25–5 MHz probe (Supersonic Imagine, Aix en Provence, France), with B-mode enabled, shear wave imaging mode enabled in penetration mode, tissue tuner 1540 m/s, dynamic range 80 dB, and using a micro manipulated probe without contact with mice. Scans were done under isoflurane anaesthesia (~ 2%, 1 l/min O_2_). Legs were shaved and mice were positioned supine on an ergometer allowing control of ankle plantar/dorsal flexion. B-mode ultrasound images and SM maps were acquired at 0°, 40°, 60°, and 80° of ankle plantar flexion (Supplementary Fig. S3A). Images were processed using MATLAB (2018b, MathWorks, Natick, MA, USA). Mean SMs in a region of interest (ROI) over the TC were extracted from SM-maps (Supplementary Fig. S3B), and the viscoelasticity index (SMi) was calculated according to Eq. (2) in the “Results” section.

### Nuclear magnetic resonance

NMR data were acquired using a 7T Bruker BioSpec system interfaced with an Avance III spectrometer (Bruker BioSpin MRI GmbH, Ettlingen, Germany). Mice were scanned under isoflurane anaesthesia (1–2%, 1 l/min O_2_) on a heating bed. Two NMR setups were used: a custom-made transceiver volume coil (300 MHz, 1 cm diameter/length) was used in the first NMR session (legs evaluated separately in consecutive exams), and a transceiver surface Cryoprobe (Bruker BioSpin MRI GmbH, Ettlingen, Germany) was used in the second session (both legs imaged simultaneously). While our surface cryoprobe allowed to image the two legs (injured and control) at the same time with an increased signal to noise ratio and spatial resolution, it is not suitable for acquisitions that require a homogeneous B1 field. Sessions including both setups were performed 1–3 days apart.

The volume coil allowed measurements of short-, intermediate-, and long-T_2_ values and fractions with an ISIS-CPMG sequence (Image selected in vivo spectroscopy—Carr-Purcell-Meiboom-Gill)^30^, and perfusion assessment with arterial spin labelling (ASL). ISIS-CPMG acquisitions require a homogeneous B1 field to ensure complete refocusing of the protons in the volume of interest in order to respect the CPMG conditions. Similarly, a spatially homogeneous tagging is required for assessing tissue perfusion using the ASL method implemented in the present work.

ISIS-CPMG data was acquired in a parallelepiped voxel placed over the tibialis cranialis muscle with mean dimensions 0.9 × 1.0 × 4.6 mm^3^ (Supplementary Fig. S2a). The inter-echo-spacing was 1 ms, 200 echoes were acquired, and the repetition time was 9.5 s.

For ASL, incoming arterial blood was saturated using a tagging module with 2000 slice-selective 90° pulses (constructor SINC10H, pulse bandwidth: 28,741 Hz, 700 µs; each pulse respecting an RF-spoiling scheme and followed by a variable spoiler gradient of 1.5 ms). The tagging module was applied during 4.6 s, to a 7.5 mm thick slice, positioned 7.5 mm proximally from the target slice centre for tagged images. Control images were acquired after applying the tagging module to a 75 mm thick slice, positioned 75 mm distally from the target slice, ensuring that the saturation slice was out of the RF-coil’s field of view. Under this scheme, control images were acquired without arterial blood saturation while keeping the frequency offset of the saturation pulse to the centre of the target slice, resulting in similar magnetization-transfer weighting in control and tagged images. After an evolution time of 200 ms, tagged or control images were acquired (single-shot RARE—rapid imaging with refocused echoes, echo-spacing: 2.05 ms, effective echo time: 2.05 ms, partial Fourier: 1.78, echo-train-length: 18, repetition time: 5 s, 2D, in plane matrix size: 32 × 32, resolution: 0.3 × 0.3 × 2 mm^3^). Tagged and control images were sequentially acquired twice, allowing the calculation of a perfusion map every 20 s. A hyperaemic response paradigm with ischemia–reperfusion stress was used to increase the global perfusion and highlight differences between normal and altered muscles^18^. Perfusion at rest was measured during 3′ 20″. Then, ischemia was induced by occlusion of the femoral artery after pulling two surgical threads placed bellow the thigh’s skin and connected to a 500 g weight. After 6′ 50″ of ischemia, the weight was removed and the tourniquet released, leading to a hyperaemic response that was monitored during 10′ 40″.

The cryoprobe setup was used in the second NMR session for: (1) high-resolution T_1_-weighted gradient-echo (GRE) images (2D-FLASH—fast low angle shot: flip angle: 6.6°, echo time: 3.4 ms, repetition time: 11.9 ms, 256 averages, resolution 50 × 50 × 200 µm^3^); (2) T_2_*-weighted images using an ultra-short time to echo (UTE) sequence^31,32^ (2D-UTE: 16 echo times: 0.012, 0.1, 0.25, 0.5, 0.6, 0.8, 1.0, 1.2, 1.4, 1.6, 1.8, 2.0, 2.6, 3.0, 6.0 and 8 ms, repetition time: 20 ms, resolution 0.2 × 0.2 × 1 mm^3^); (3) T_2_-maps (2D-MSME—multi-slice-multi-echo: repetition time: 3500 ms, first echo time: 5.15 ms, echo spacing: 5.15 ms, 32 echoes, resolution 0.1 × 0.1 × 1 mm^3^) and (4) T_1_-maps (Saturation-recovery 2D-RARE: adiabatic saturation pulse, echo time: 6.66 ms, 13 repetition times: 82.9, 100, 128, 171, 214, 602, 286, 359, 1010, 1694, 2842, 4769, 8000 ms, muscle MRI: resolution 0.1 × 0.1 × 1 mm^3^). T_1_-maps were acquired before (native-T_1_) and one hour after the intraperitoneal injection of a Gd-contrast agent (Gd-CA) (Dotarem, Guerbet, Villepinte, France; 2 µmol Gd/g mouse weight, saline:Dotarem 1.6:1). Gd-CA was injected through an intraperitoneal catheter placed before the exam and filled with saline solution (NaCl 0.9%, 100–150 µl). Blood samples were collected using heparinized capillaries immediately before the second NMR session and after the last T_1_-map. Samples were centrifuged (1500*g*, 15′, 4 °C) and kept at 4 °C before T_1_ measurements were performed (up to 2 h). After samples reached room temperature, plasma-T_1_ pre- and post-Gd-CA was measured with the same T_1_-mapping sequence used in vivo, using lower spatial resolution (plasma MRI: resolution 0.2 × 0.2 × 4 mm^3^). Muscle and plasma T_1_ measurements were then combined for estimation of ECV^6^ (Supplementary Fig. S1).

### NMR data processing

Python (https://www.python.org/) and MATLAB custom routines were used to process NMR data.

Muscle and plasma T_1_ maps were estimated by fitting T_1_ recovery curves to a mono-exponential model accounting for a variable flip angle. ECV maps were estimated by combining muscle and plasma T_1_ values before and one hour after Gd-CA injection, according to Eq. (1). Supplementary Fig. S1 shows representative T_1_ maps for one mouse before and after Gd-CA injection in vivo and in plasma samples, as well as the correspondent ECV map.

Two complementary approaches were used to estimate T_2_ values: MSME and ISIS-CPMG. MSME data allowed the estimation of T_2_ maps by matching the signal from MSME images in each voxel to a dictionary of normalized signal decay curves (variable T_2_ and B_1_ values), generated with a single-component extended phase-graph (EPG) algorithm^33^. B_1_ values were allowed to vary in the model to accommodate the expected differences in flip angle with the distance to the surface coil. Mean T_1_, ECV and T_2_ values from the respective maps were extracted from regions of interest (ROIs) drawn over left (injured) and right (control) tibialis cranialis (Supplementary Fig. S2b).

ISIS-CPMG data allowed the estimation of multiple T_2_ values and fractions due to the higher signal-to-noise ratio and finer temporal sampling of the signal decay than MSME data, at the cost of lower spatial resolution and coverage. T_2_ values and fractions were estimated by fitting the ISIS-CPMG signal decay to a mono-exponential (Eq. 3), a bi-exponential (Eq. 4) and a tri-exponential (Eq. 5) decay models:

3 $$S_{m} = M_{0} *\exp \left( { - {\raise0.7ex\hbox{${TE}$} \!\mathord{\left/ {\vphantom {{TE} {T_{2} }}}\right.\kern-\nulldelimiterspace} \!\lower0.7ex\hbox{${T_{2} }$}}} \right)$$

4 $$S_{b} = M_{0s} *\exp \left( { - {\raise0.7ex\hbox{${TE}$} \!\mathord{\left/ {\vphantom {{TE} {T_{2s} }}}\right.\kern-\nulldelimiterspace} \!\lower0.7ex\hbox{${T_{2s} }$}}} \right) + M_{0l} *\exp \left( { - {\raise0.7ex\hbox{${TE}$} \!\mathord{\left/ {\vphantom {{TE} {T_{2l} }}}\right.\kern-\nulldelimiterspace} \!\lower0.7ex\hbox{${T_{2l} }$}}} \right)$$

5 $$S_{t} = M_{0s} *\exp \left( { - {\raise0.7ex\hbox{${TE}$} \!\mathord{\left/ {\vphantom {{TE} {T_{2s} }}}\right.\kern-\nulldelimiterspace} \!\lower0.7ex\hbox{${T_{2s} }$}}} \right) + M_{0i} *\exp \left( { - {\raise0.7ex\hbox{${TE}$} \!\mathord{\left/ {\vphantom {{TE} {T_{2i} }}}\right.\kern-\nulldelimiterspace} \!\lower0.7ex\hbox{${T_{2i} }$}}} \right) + M_{0l} *\exp \left( { - {\raise0.7ex\hbox{${TE}$} \!\mathord{\left/ {\vphantom {{TE} {T_{2l} }}}\right.\kern-\nulldelimiterspace} \!\lower0.7ex\hbox{${T_{2l} }$}}} \right)$$

where the indexes *s*, *i* and *l* refer to short-, intermediary- and long-T_2_ or T_2_-fraction, respectively.

Fitting results were compared with F-tests and the tri-exponential model (Eq. 5) was the one that best fitted the majority of the data. This model was then selected for the statistical analysis of T_2_ and respective fractions (short-, intermediate-, and long-T_2_). Supplementary Fig. S2a illustrates the voxel positioning over the tibialis cranialis muscle, and the corresponding signal fitted to a tri-exponential decay.

T_2_* values and fractions were estimated by fitting the UTE amplitude signal decay from ROIs over injured and control muscles to three models: a mono-exponential decay (Eq. 6), a bi-exponential decay with two water components with short- and long-T_2_* (Eq. 7), and a bi-exponential decay model with a short-T_2_* component chemically shifted by 970 Hz from the water frequency in addition to a water component with long-T_2_* (Eq. 8)^10,21,22^:

6 $$S_{m} = A_{0} *\exp \left( { - {\raise0.7ex\hbox{${TE}$} \!\mathord{\left/ {\vphantom {{TE} {T_{2}^{*} }}}\right.\kern-\nulldelimiterspace} \!\lower0.7ex\hbox{${T_{2}^{*} }$}}} \right)$$

7 $$S_{b} = A_{short} *\exp \left( { - {\raise0.7ex\hbox{${TE}$} \!\mathord{\left/ {\vphantom {{TE} {T_{2short}^{*} }}}\right.\kern-\nulldelimiterspace} \!\lower0.7ex\hbox{${T_{2short}^{*} }$}}} \right) + A_{long} *\exp \left( { - {\raise0.7ex\hbox{${TE}$} \!\mathord{\left/ {\vphantom {{TE} {T_{2long}^{*} }}}\right.\kern-\nulldelimiterspace} \!\lower0.7ex\hbox{${T_{2long}^{*} }$}}} \right)$$

8 $$S_{osc} = A_{short} *\exp \left[ {\left( { - {\raise0.7ex\hbox{$1$} \!\mathord{\left/ {\vphantom {1 {T_{2short}^{*} }}}\right.\kern-\nulldelimiterspace} \!\lower0.7ex\hbox{${T_{2short}^{*} }$}} + 970 \cdot 2\pi i} \right) \cdot TE} \right] + A_{long} *\exp \left( { - {\raise0.7ex\hbox{${TE}$} \!\mathord{\left/ {\vphantom {{TE} {T_{2long}^{*} }}}\right.\kern-\nulldelimiterspace} \!\lower0.7ex\hbox{${T_{2long}^{*} }$}}} \right)$$

where, ${A}_{0}$ is the total magnetization at steady state, ${A}_{short}$ is the short-T_2_* signal amplitude at TE = 0, and ${A}_{long}$ is the water long-T_2_* signal amplitude at TE = 0 ms. Models were limited to up to two components to avoid overfitting. Results after fitting the signal decay to each model were compared with F-tests. The bi-exponential model with an oscillatory component (Eq. 8) presented the highest F-value in the majority of the data and was then selected for statistical analysis. Supplementary Fig. S2c shows representative UTE images at different echo times, the corresponding signal decay in the ROI and the curve fitted using the bi-exponential model with an oscillatory component.

Muscle perfusion in ROIs drawn over injured and control muscles was calculated from the relative difference between consecutive tagged (${M}^{+}$) and control ($M$) images, according to Eq. (9):

9 $$f = {\raise0.7ex\hbox{$\lambda $} \!\mathord{\left/ {\vphantom {\lambda {T1_{m} }}}\right.\kern-\nulldelimiterspace} \!\lower0.7ex\hbox{${T1_{m} }$}}*\left[ {\frac{{{{(M - M^{ + } )} \mathord{\left/ {\vphantom {{(M - M^{ + } )} M}} \right. \kern-\nulldelimiterspace} M}}}{{\exp \left( { - {\raise0.7ex\hbox{${tT}$} \!\mathord{\left/ {\vphantom {{tT} {T1_{a} }}}\right.\kern-\nulldelimiterspace} \!\lower0.7ex\hbox{${T1_{a} }$}}} \right) - {{(M - M^{ + } )} \mathord{\left/ {\vphantom {{(M - M^{ + } )} M}} \right. \kern-\nulldelimiterspace} M}}}} \right]$$

where $\lambda$ is the tissue-blood partition coefficient, estimated as 0.75 for skeletal muscle; ${T1}_{m}$ is the intrinsic muscle T_1_ (in absence of perfusion), estimated as 2 s since it should be higher than the T_1_ value of muscles under perfusion (1649.9 (37.6) ms in this study); ${T1}_{a}$ is the intrinsic arterial blood T_1_, set as 2.2 s^34^; and $tT$ is the mean travel time of arterial blood from the tagging slice to the imaged voxel (3.5 mm away), estimated as 0.2 s. Total perfusion, integrated over the recovery time, and maximal perfusion were then extracted. Although there is no consensus on the precise values for the tissue:blood partition coefficient, intrinsic muscle T_1_ and arterial blood T_1_, in this study these values were used as scaling factors to estimate the tissue perfusion in ml(plasma)/min/100 ml(tissue) from ASL measurements. Any difference observed in perfusion measurements between groups is therefore independent of the choice of these values.

Image texture analysis was performed in T_1_-weighted high spatial resolution images through histogram features. Skewness, kurtosis, and energy were extracted from the same ROIs as for muscle T_1_, T_2_, and ECV (Fig. 5). Energy reflects the uniformity of the signal distribution, and was defined as:

10 $$energy=\sum_{i}{{p}_{i}}^{2},$$

where *p*_*i*_ refers to the proportion of pixel with signal intensity *i*.

### Histology

Up to 48 h after NMR, both tibialis cranialis were collected, fixed in formalin 10% during 48 h and kept in ethanol 70% before the preparation of paraffin blocks. Three slices per muscle (¼, ½, and ¾ of muscle length) were stained with Sirius red and haematoxylin–eosin. Slides were digitalised using Hamamatsu NanoZoomer 2.0 HT digital slide scanner and visualized using the software NDP.view2 (Hamamatsu Photonics K.K., Hamamatsu, Japan). Collagen fraction was quantified from Sirius red slides with Fiji (NIH, Bethesda, MD^35^). Endomysial collagen was quantified in ROIs drawn excluding tendons, fascia and perivascular connective tissue around veins or arteries. The degree of inflammation, centronucleation, fat infiltration and necrotic calcifications in haematoxylin–eosin slides was rated independently by two researchers with more than 10 years of experience in muscle histology (ABMB, YF). Each of these variables was scored as absent, mild, moderate or severe. A stringent classification of inflammation was adopted because even very mild alterations could affect NMR and SWE metrics. Inflammation was considered absent when no sign of infiltrated inflammatory cells or necrosis were observed; mild when punctual infiltration of inflammatory cells was detected; moderate when scattered infiltration of immune cells and possible necrosis was observed, but in a reduced level; and severe when clear infiltration of inflammatory cells and presence of necrotic muscle fibers were detected. Calcifications and fat infiltration were scored based on the number and size of calcified fibers or adipose cells clusters. Centronucleation was scored as absent when central nuclei were observed in less than 1% of fibers; mild when 2–30% of myofibers had central nuclei; moderate when 30–60% of myofibers had central nuclei; and severe when more than 60% of myofibers had central nuclei. Scores were compared and a consensus was reached in case of discordances.

### Statistical analysis

Injured and control muscles were compared for each strain and after grouping C57BL/6 and DBA/2J mice. Grouping the strains allowed to increase the sample size and improved the statistical power of the comparisons. Additionally, it allowed to cover a broader range of collagen fractions, improving the identification of correlations between collagen fraction and NMR or SWE metrics.

Statistical analysis was performed using R (3.5.1; R Core Team, 2018)^36^ and RStudio environment (1.1.463, RStudio, Boston, MA, USA)^37^, and the packages dplyr^38^, Hmisc^39^, psych^40^, and stats^36^. Figures were generated using the packages ggplot2^41^, ggpubr^42^, patchwork^43^, plotROC^44^, pROC^45^, unikn^46^ and viridis^47^. Kruskal–Wallis and Wilcoxon post-hoc tests were used for multiple comparisons. Paired-Wilcoxon tests were used for comparisons between injured and control muscles. Bonferroni correction accounted for multiple comparisons. Spearman rank-order correlations and partial correlations were calculated between NMR/SWE variables and total/endomysial collagen fractions. Inflammation, centronucleated fibers, calcifications, maximal and total perfusion accounted as covariates in partial correlations. Statistical significance was set at *p* < 0.05. Numerical data are reported as median (inter-quartiles range). Delta is the difference between injured and control muscles for each animal, reported as median (inter-quartile range).

## Supplementary Information

Supplementary Information.

## Data Availability

The datasets generated and analysed during the current study are available from the corresponding author on reasonable request.
