# Supplementary material for: Non-invasive assessment of skeletal muscle fibrosis in mice using nuclear magnetic resonance imaging and ultrasound shear wave elastography
Source: Sci Rep. 2021 Jan 11;11:284. doi: 10.1038/s41598-020-78747-8 (PMC7801669; doi:10.1038/s41598-020-78747-8)
Supplement: Supplementary file 1 — Supplementary Information. [file 41598_2020_78747_MOESM1_ESM.pdf]

Title:

Non-invasive assessment of skeletal muscle fibrosis in mice using nuclear magnetic resonance imaging and ultrasound shear wave elastography

Authors:

Aurea B. Martins-Bach[1], Damien Bachasson[2], Ericky C. A. Araujo[1], Lucas Soustelle[3], Paulo Loureiro de Sousa[3], Yves Fromes[1], Pierre G. Carlier[1]

[1] AIM & CEA NMR Laboratory, Neuromuscular Investigation Center, Institute of Myology, Paris, France

[2] Neuromuscular Physiology Laboratory, Neuromuscular Investigation Center, Institute of Myology, Paris, France

[3] Université de Strasbourg, CNRS, ICube, FMTS, Strasbourg, France

Supplementary Figure S1.

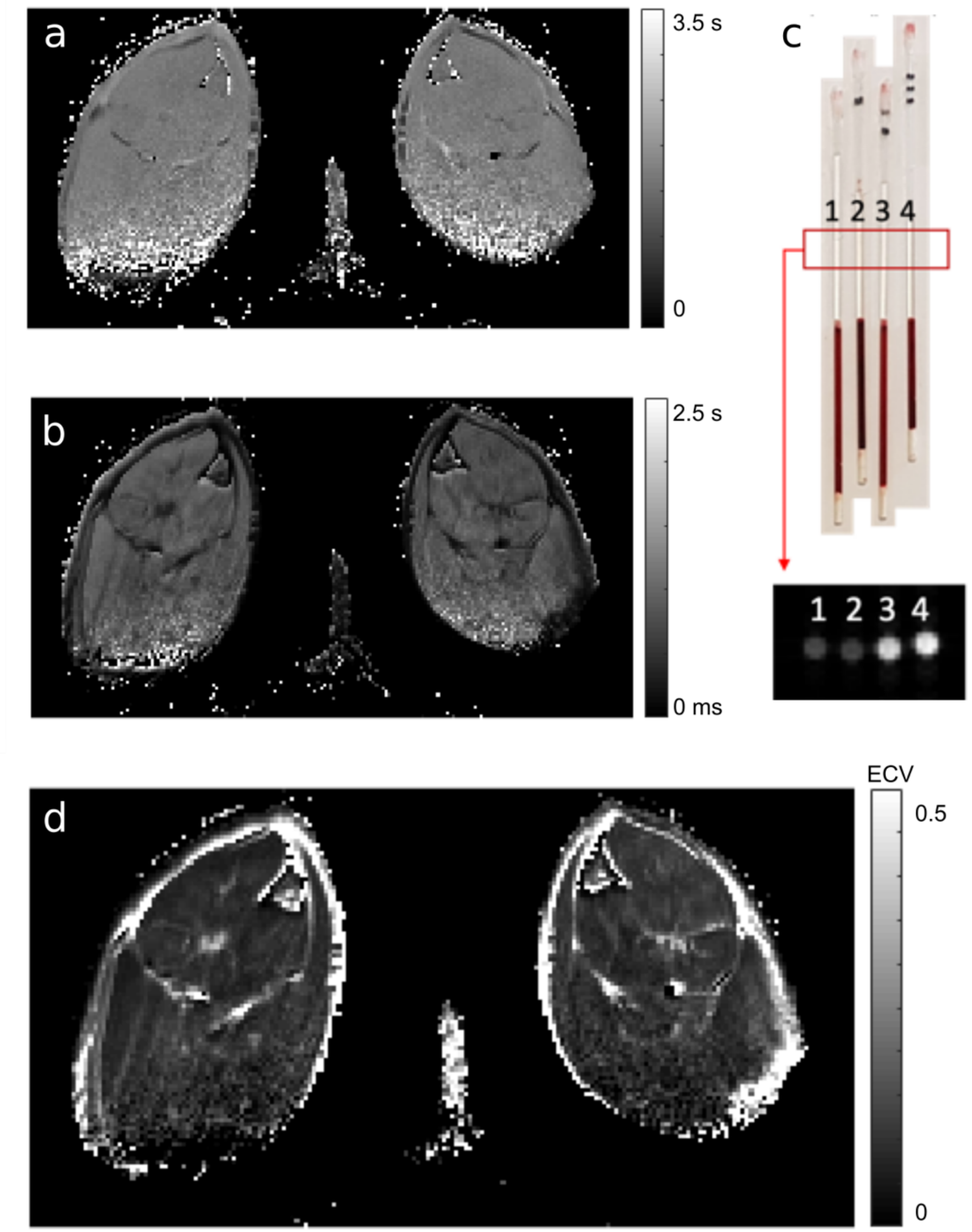

Supplementary Figure S1. *Representative muscle and plasma T_1 maps, and estimated ECV map*. (a) Native T_1 map (before injection of Gd-CA) estimated from *in vivo* imaging of mouse legs. (b) T_1 map from the same animal, 1-hour after intraperitoneal injection of Gd-CA. (c) Blood samples were collected by tail punction before MRI (samples 1-2) and after the last T_1-map post Gd-CA injection (samples 3-4). Samples

were centrifuged, and plasma was imaged using the same protocol as for in vivo T_1 mapping (bottom). Samples were taken in duplicate. (d) ECV map, estimated from T_1 values pre- and post- Gd-CA injection for tissue and plasma.

Supplementary Figure S2.

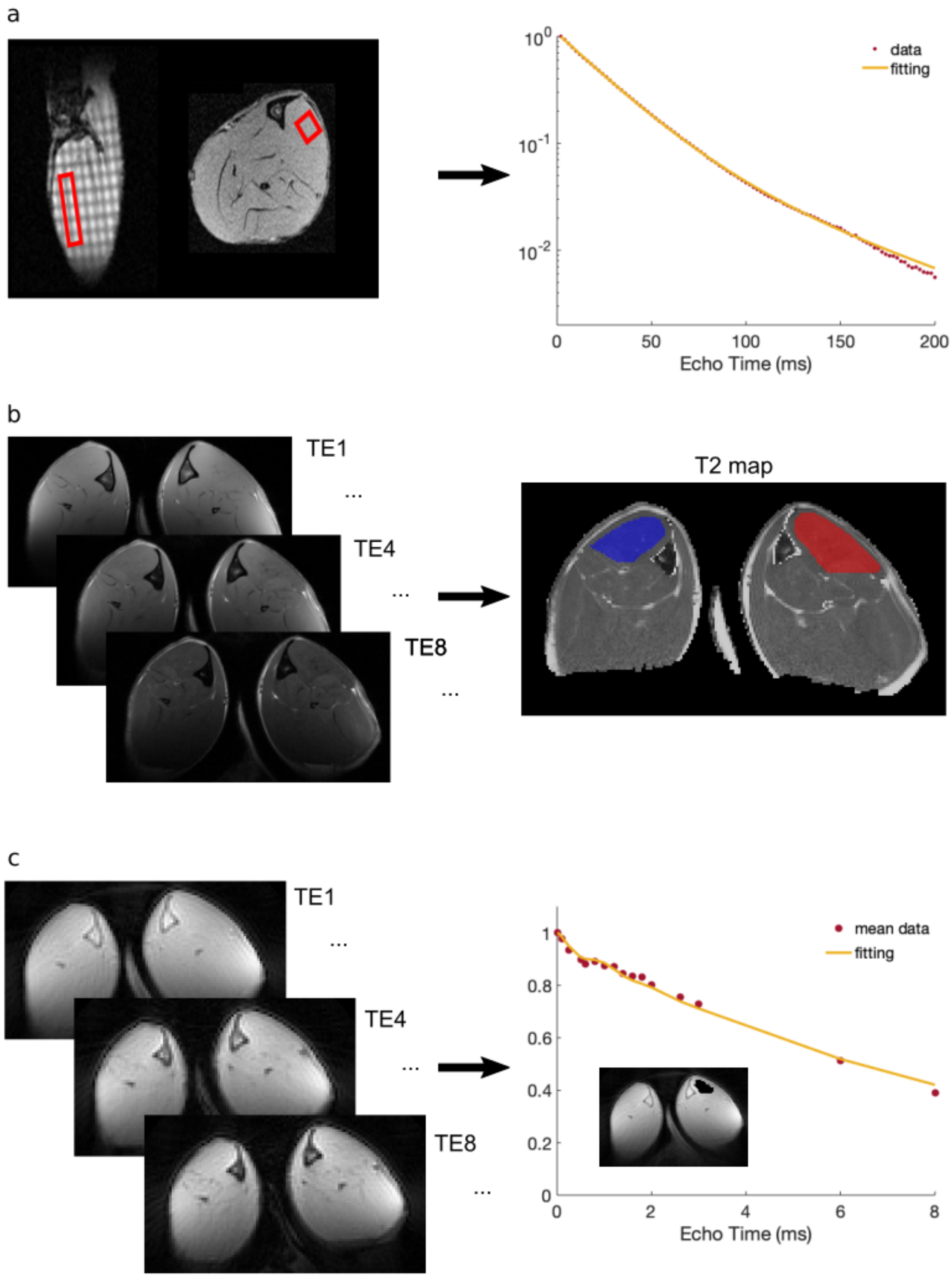

Supplementary Figure S2. *Representative images and ROI selection for T_2 and T_2* measurements*. (a) ISIS-CPMG signal was acquired from a volume positioned over the tibialis cranialis muscle (red parallelepiped, sagittal and axial views). On the right, ISIS-CPMG signal decay as a function of echo time (red dots), and the fitted tri-exponential model (orange line). (b) Representative images acquired with MSME at different echo times (left, TE1 = 5.15 ms, TE4 = 20.6 ms, TE8 = 41.2 ms), and the corresponded T_2 map (right). Two ROIs were draw over left (red) and right (blue) tibialis cranialis. (c) Representative images acquired with the UTE sequence, at different echo times (left, TE1 = 0.012 ms, TE4 = 0.5 ms, TE8 = 1.2 ms). The mean signal in ROIs draw over each tibialis cranialis was fitted to a bi-exponential decay with an off-resonance component (right).

Supplementary Figure S3.

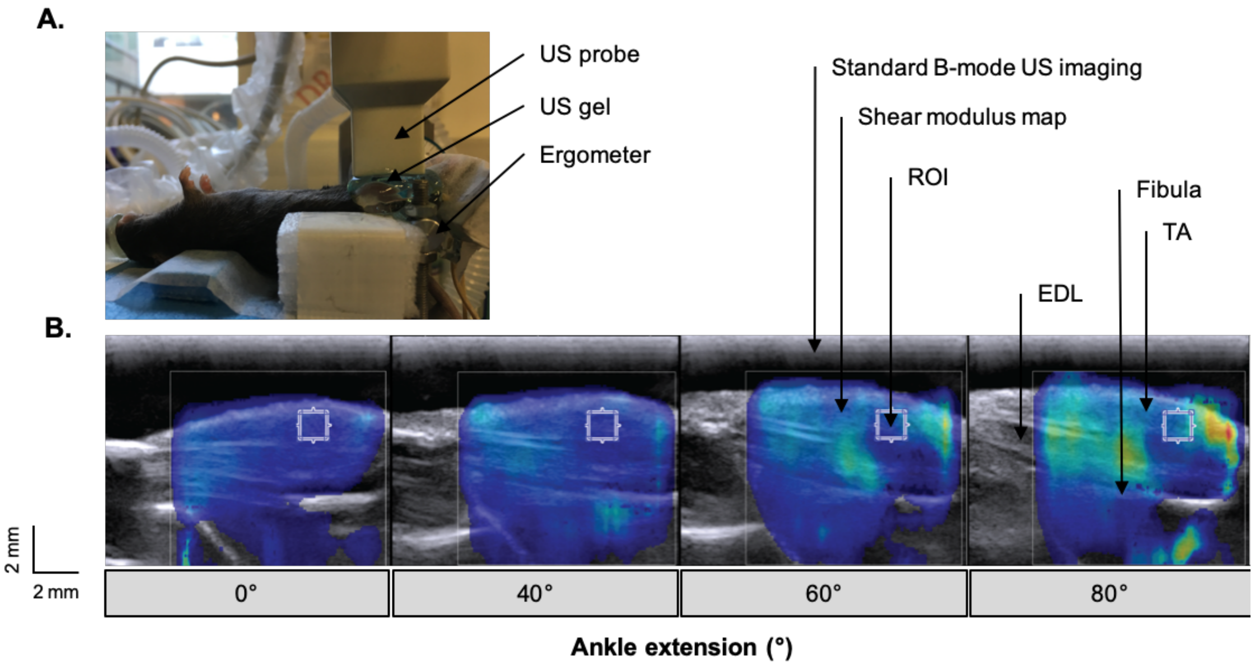

Supplementary Figure S3. *Experimental setup for shear-wave elastography*. (A) Mouse and probe positioning for standardized ultrasound shear-modulus measurements. (B) Shear-modulus and standard B-mode ultrasound images were acquired at different ankle extension positions, to allow dynamic assessment of muscle viscoelastic properties. Mean SM at each angle was extracted by placing a square region of interest (ROI) within the proximal third portion of the tibialis cranialis.

Supplementary Table S1: Correlations and partial correlations between collagen fraction and non-invasive NMR and SWE metrics.

Modality & Metric	Correlation							Partial correlation					
	Total collagen			Endomysial collagen				Total collagen			Endomysial collagen		
	R	*p*		*R*	*p*			*R'*	*p*		*R'*	*p*	
T_1-maps													
native-T_1	0.30	0.079		0.18	0.281		*native-T_1*	0.29	0.097		0.17	0.357	
ECV	**0.50**	**0.002**	**	**0.46**	**0.005**	**	***ECV***	**0.38**	**0.028**	*	0.30	0.085	
T_2-map													
MRI-T_2	**-0.48**	**0.003**	**	**-0.37**	**0.027**	*	***T_2***	**-0.50**	**0.003**	**	**-0.36**	**0.042**	*
CPMG													
T_2s	-0.14	0.399		-0.14	0.422		*T_2s*	-0.10	0.587		-0.08	0.649	
T_2i	-0.04	0.838		-0.03	0.874		*T_2i*	-0.21	0.232		-0.20	0.262	
T_2l	-0.13	0.435		-0.16	0.366		*T_2l*	-0.21	0.238		-0.22	0.215	
T_2s-Fr	-0.14	0.402		-0.13	0.447		*T_2s-Fr*	-0.28	0.113		-0.24	0.186	
T_2i-Fr	-0.10	0.548		-0.04	0.833		*T_2i-Fr*	-0.08	0.671		0.00	0.988	
UTE													
*T_2*s-Fr*	-0.01	0.937		-0.08	0.661		*T_2*s-Fr*	-0.07	0.679		-0.19	0.277	
T_2-short*	0.18	0.306		0.21	0.213		***T_2*-short***	0.26	0.144		**0.36**	**0.038**	*
T_2-long*	-0.15	0.383		-0.25	0.148		*T_2*-long*	0.10	0.589		-0.05	0.776	
High-res T_1w													
Skewness	-0.20	0.254		-0.12	0.500		*Skewness*	-0.11	0.542		0.01	0.952	
Kurtosis	**0.33**	**0.046**	*	**0.42**	**0.010**	*	***Kurtosis***	0.28	0.121		**0.36**	**0.039**	*
Energy	0.22	0.206		**0.35**	**0.037**	*	*Energy*	0.13	0.460		0.28	0.116	
SWE													
SM_0	0.06	0.747		0.09	0.622		***SM_0***	0.31	0.076		**0.38**	**0.031**	*
SM_{40}	0.06	0.730		0.09	0.632		*SM_{40}*	-0.06	0.748		-0.02	0.919	
SM_{60}	0.16	0.390		0.16	0.388		*SM_{60}*	0.06	0.748		0.07	0.711	

SM_{80}	**0.46**	**0.009**	**	**0.45**	**0.011**	*	***SM_{80}***	**0.35**	**0.044**	*	**0.35**	**0.047**	*
SMi	**0.41**	**0.019**	*	**0.35**	**0.049**	*	***SMi***	0.21	0.233		0.13	0.479	

*T_2s-Fr: Fraction of the signal with short-T_2 in CPMG data; T_2i-Fr: fraction of the signal with intermediary-T_2 in CPMG; T_2s, T_2i, T_2l: short-, intermediary- and long- T_2, estimated from ISIS-CPMG data. SWE: shear wave elastography; SM: shear modulus at different angles of plantar flexion; SMi: viscoelastic index. Significant correlations are highlighted in bold. *$p<0.05$, **$p<0.01$*

Supplementary Table S2: T_2 values and fractions estimated from ISIS-CPMG data

	Injured	Control	Paired difference	p-value
T_2s				
all	2.4 (3.5) ms	4.2 (7.0) ms	-2.0 (5.7) ms	0.17
DBA/2J	1.0 (2.7) ms	4.0 (5.3) ms	-1.2 (4.7) ms	0.41
C57BL/6	3.1 (3.2) ms	5.6 (5.5) ms	-3.2 (4.9) ms	0.22
T_2s fraction				
all	5.3 (3.3) %	5.5 (8.0) %	-1.3 (8.8) %	0.64
DBA/2J	5.6 (9.2) %	6.0 (7.1) %	-3.3 (10.9) %	1.00
C57BL/6	5.1 (2.0) %	5.0 (5.8)%	-0.5 (8.2) %	0.81
T_2i				
all	25.4 (1.9) ms	24.7 (1.5) ms	0.7 (2.6) ms	0.37
DBA/2J	24.6 (2.1) ms	24.7 (1.4) ms	1.0 (2.7) ms	0.32
C57BL/6	25.6 (0.9) ms	24.6 (2.2) ms	0.4 (2.4) ms	0.94
T_2i fraction				
all	89.2(6.5) %	88.2(6.2) %	1.1(8.7) %	0.58
DBA/2J	86.9 (4.3) %	86.5 (5.9) %	0.9 (7.8) %	0.83
C57BL/6	91.1 (4.8) %	89.8 (5.3) %	4.0 (9.4) %	0.47
T_2l				
all	97.7 (69.0) ms	103.1 (30.7) ms	2.0 (74.8) ms	0.87
DBA/2J	127.3 (70.3) ms	103.1 (25.1) ms	13.4 (71.2) ms	0.46
C57BL/6	96.7 (26.1) ms	115.0 (68.5) ms	-9.3 (101.7) ms	0.69
T_2l fraction				
all	3.5 (4.0) %	4.3 (2.3) %	-0.4 (6.6) %	0.90
DBA/2J	4.3 (4.2) %	4.4 (1.7) %	-0.4 (5.8) %	0.76
C57BL/6	2.8 (3.1) %	3.2 (3.1) %	-0.4 (5.9) %	0.81

T_2s, T_2i, T_2l: short-, intermediary- and long-T_2 estimated from ISIS-CPMG data fitted to a tri-exponential model.

Supplementary Table S3: Texture analysis using histogram features from T_1-weighted high resolution images.

	Injured	Control	Paired difference	p-value
Skewness				
all	0.34 (0.85)	0.56 (0.80)	-0.35 (1.11)	0.26
DBA/2J	0.36 (1.14)	0.46 (0.73)	-0.20 (1.29)	0.37
C57Bl/6	0.34 (0.81)	0.84 (0.39)	-0.49 (1.12)	0.81
Kurtosis				
all	4.7 (2.8)	3.6 (0.7)	1.57 (3.35)	0.03 *
DBA/2J	4.6 (2.7)	3.5 (0.7)	1.66 (3.26)	0.17
C57Bl/6	5.3 (2.8)	3.9 (0.6)	1.49 (3.19)	0.11
Energy (x10^3)				
all	11.1 (3.2)	9.5 (2.5)	1.4 (4.0)	0.02 *
DBA/2J	10.8 (3.0)	9.6 (3.1)	1.0 (4.2)	0.24
C57Bl/6	13.3 (3.4)	9.4 (1.7)	1.8 (4.0)	0.015 *

*Data is presented as median (inter-quartile range). *: p<0.05*

Supplementary Table S4: Shear modulus (SM) at different plantar flexion angles and viscoelastic index in fibrotic (injured) and normal muscle.

	Injured	Control	Paired difference	p-value
SM_0 (kPa)				
all	24.7 (7.1)	27.0 (8.0)	-2.7 (8.6)	0.23
DBA/2J	25.3 (8.1)	29.5 (5.7)	-4.2 (3.7)	0.02 *
C57BL/6	24.7 (4.2)	23.2 (5.2)	3.2 (6.4)	0.56
SM_{40} (kPa)				
all	33.9(4.9)	30.0(7.9)	3.7(11.7)	0.35
DBA/2J	32.8 (7.3)	33.9 (10.0)	-1.0 (10.3)	0.70
C57BL/6	34.8 (2.5)	27.8 (1.0)	5.8 (5.9)	0.03 *
SM_{60} (kPa)				
all	46.6(11.2)	38.8(11.7)	4.7(16.4)	0.13
DBA/2J	46.4 (15.5)	44.0 (14.7)	0.3 (22.3)	0.77
C57BL/6	44.0 (10.5)	34.0 (3.4)	10.2 (12.5)	0.03 *
SM_{80} (kPa)				
all	54.4(16.0)	44.6(21.5)	8.3(24.9)	0.07
DBA/2J	62.5 (18.2)	54.5 (20.3)	3.1 (31.6)	0.49
C57BL/6	49.3 (7.5)	36.0 (9.3)	13.4 (6.8)	0.06
SM_i (a.u.)				
all	1.17(0.99)	0.67(0.44)	0.29(0.86)	0.02 *
DBA/2J	1.55 (0.93)	0.93 (0.39)	0.35 (1.08)	0.11
C57BL/6	0.73 (0.35)	0.58 (0.32)	0.23 (0.54)	0.16`

SM_i is the normalized difference between SM at 80° (SM_{80}) and SM at rest (SM_0). **: $p<0.05$ in paired comparisons between injured and control muscles in the same mouse.*
